# Supplementary material for: Fourier transform spectrometer on silicon with thermo-optic non-linearity and dispersion correction
Source: Nat Commun. 2018 Feb 14;9:665. doi: 10.1038/s41467-018-03004-6 (PMC5813211; doi:10.1038/s41467-018-03004-6)
Supplement: Supplementary file 1 — Supplementary information [file 41467_2018_3004_MOESM1_ESM.pdf]

Fourier transform spectrometer on silicon with thermo-optic non-linearity and dispersion correction

Souza *et al.*

Supplementary Note 1 - Dispersion, thermo-optic and thermal expansion parameters

In this Supplementary Note, we present the properties of the silicon-on-insulator (SOI) waveguides used in our realization of the chip-scale silicon photonics Fourier Transform spectrometer (Si-FTS). Our design exploits the quasi-TE mode of a 250×550 nm^2 SOI waveguide. The effective index, its dispersion and its thermo-optic derivatives are summarized in Supplementary Table 1. These coefficients were obtained from finite difference element (FDE) simulations in the frequency range from 180 THz to 210 THz and for temperatures between 300 K and 400 K. The simulations were performed using the FDE solver Lumerical MODE with modified bulk refractive index models for both the silicon core and the silica cladding. The models include a Sellmeier dependence for both silicon[1] and silica[2]. The refractive index models presented in these references also include temperature dependence in the range from 20 K to 300 K, whereas we are interested in the temperature range from 300 K to 400 K. For the silica cladding, the thermo-optic coefficient (TOC) is practically constant around 300 K ($3 \cdot 10^{-5}$ K^{-1}), and it is safe to use the thermo-optic model provided in[2]. Silicon's TOC, on the other hand, varies significantly with temperature and the model provided in[1] does not deliver consistent results in the temperature range of interest. For this reason, we consider an index model for silicon that combines the dispersion from[1] with the TOC obtained from investigations comprising temperatures from 300 K to 400 K[3]. It is safe to introduce the thermo-optic behavior as independent additional terms in the refractive index model since the crossed-dependence $\frac{\partial^2 n_{\mathrm{Si}}}{\partial \nu \partial T}$ is negligible in the frequency and temperature ranges of interest[4].

The thermo-optic behavior of silicon around 300 K is presented in Supplementary Figure 1. In this model, the TOC has a second-order dependence with temperature (Supplementary Figure 1a) and the contribution of the temperature dependent terms with respect to the zeroth order is depicted in Supplementary Figure 1b. It shows that the first order has a non-negligible contribution reaching close to 14% of the zeroth order for a temperature change of 100 K, while the second order reaches a maximum of 1% in the same temperature range and can be neglected in practice.

In addition to the thermo-optic effect, thermal expansion changes the total length of the interferometer's arm and must be accounted for. The thermal expansion coefficient of silicon around 300 K is presented in Supplementary Figure 2a and presents a strong dependence with temperature[5]. The

**Parameter**	**Value**	**Unit**	**Parameter**	**Value**	**Unit**
$n_{\mathrm{eff}}\rvert_{\nu_0}$	2.62	—	$\partial_{T^2} n$	7.0×10^{-7}	K^{-2}
$\partial_{\nu} n$	7.8×10^{-3}	THz^{-1}	$\partial_{\nu,T^2} n$	-3.7×10^{-10}	K^{-2} THz^{-1}
$\partial_{\nu^2} n$	$- 9.0 \times 10^{-4}$	THz^{-2}	$\partial_{\nu^2,T^2} n$	-3.7×10^{-12}	K^{-2} THz^{-2}
$\partial_{\nu^3} n$	1.6×10^{-6}	THz^{-3}	$\partial_{\nu^3,T^2} n$	4.6×10^{-15}	K^{-2} THz^{-3}
$\partial_{T} n$	1.85×10^{-4}	K^{-1}	α_1	2.5×10^{-6}	K^{-1}
$\partial_{\nu,T} n$	2.1×10^{-7}	K^{-1} THz^{-1}	α_2	8.5×10^{-9}	K^{-2}
$\partial_{\nu^2,T} n$	-2.0×10^{-9}	K^{-1} THz^{-2}	α_3	-2.3×10^{-11}	K^{-3}
$\partial_{\nu^3,T} n$	1.8×10^{-10}	K^{-1} THz^{-3}			

Supplementary Table 1: Dispersion, thermo-optic, and thermal expansion parameters. The dispersion and thermo-optic coefficients are obtained for the quasi-TE mode of a 250×550 nm^2 SOI strip waveguide at the telecom band ($\nu_0 = 193.414$ THz). A contracted notation for partial derivatives is used, $\frac{\partial n_{\mathrm{eff}}}{\partial x} \equiv \partial_x n$.

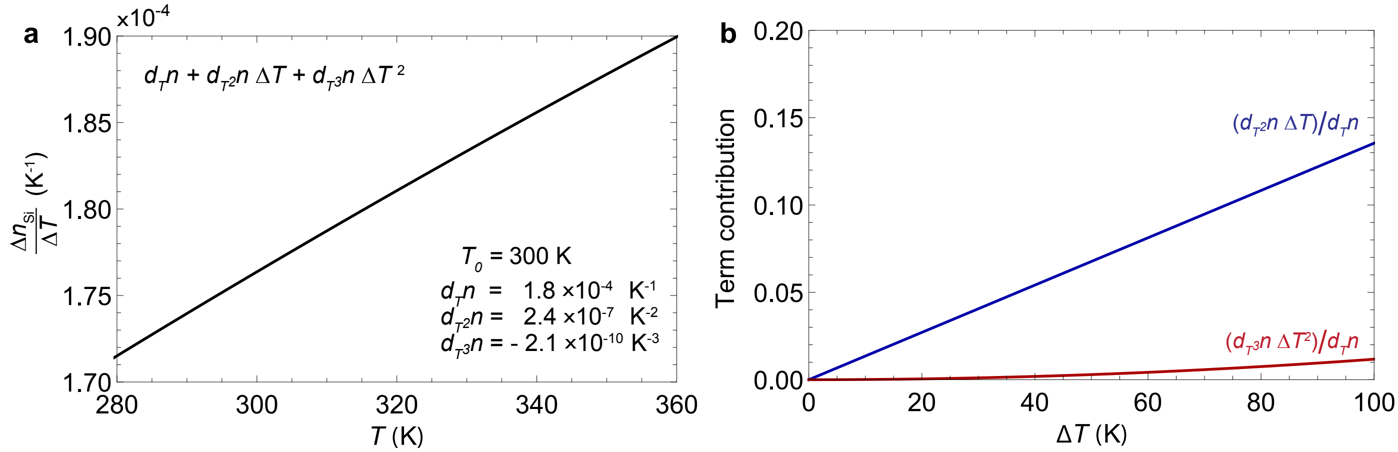

Supplementary Figure 1: Thermo-optic coefficient (TOC) of silicon. **a.** Around 300 K, the TOC has a second order dependence with temperature with coefficients shown in the figure. **b.** Contribution of first and second order terms with respect to zeroth order. For a temperature change of 100 K, the first order contributes around 14%. The second order contribution is around 1% and can be neglected. The model is based on[3].

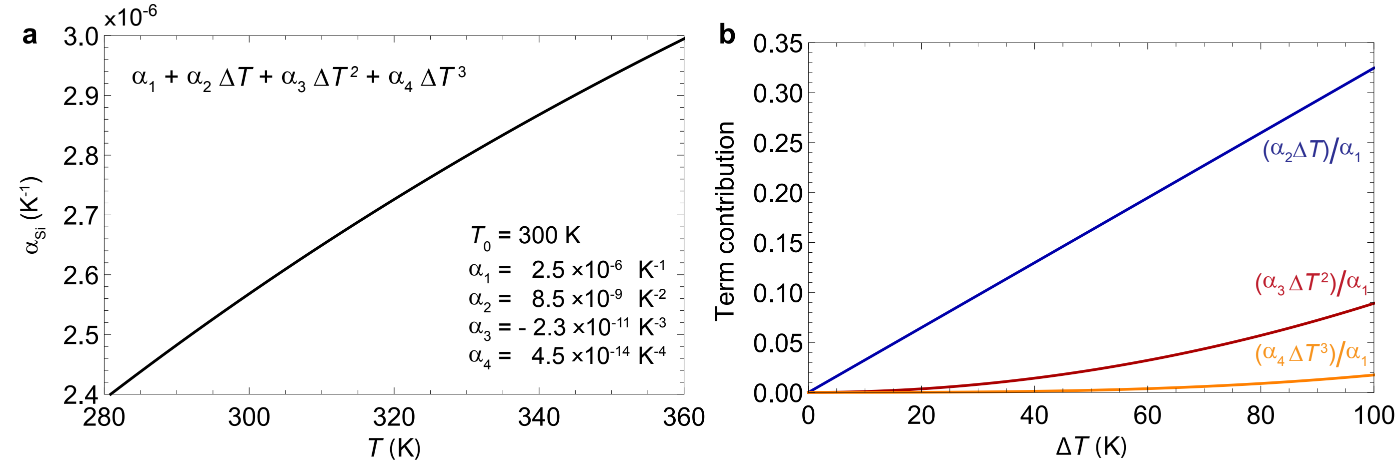

Supplementary Figure 2: Thermal expansion coefficient of silicon. **a.** Around 300 K it has a strong dependence with temperature, modeled using a third order polynomial with the coefficients shown in the figure. **b.** Contribution of first, second and third order terms with respect to zeroth order. For a temperature change of 100 K, the first and second order contribute around 33% and 10%. The third order contribution is around 2% and can be neglected. The model is based on[5].

temperature dependence is well described by a third-order polynomial with the coefficients presented as insets in Supplementary Figure 2a. The contribution of the temperature-dependent terms with respect to the zeroth order in depicted in Supplementary Figure 2b. The first and second order terms contribute to 33% and 10% of the zeroth order value for temperature changes of 100 K. The third order reaches a maximum of 2% in the same temperature range and can be neglected. The coefficients used in our model (zeroth, first and second order) are also summarized in Supplementary Table 1.

Supplementary Note 2 - Interferogram and power spectral density for the Si-FTS

In this section we derive a Fourier Transform (FT) relation between the varying optical power at the output of a Mach-Zehnder interferometer (MZI), I_{out}, and the power spectral density of a broadband source, $PSD(\nu)$. In the ideal FTS the kernel of the FT is a complex exponential with argument $2\pi\nu\tau$, where ν is the optical frequency and τ is the time delay between the two arms of the MZI. We show that for the Si-FTS the kernel can still be presented with an argument of the form $2\pi\times$*frequency*$\times$*delay*, provided that *frequency* and *delay* are modified to account for dispersion, thermo-optic non-linearity and thermal expansion. In this general treatment, fabrication imperfections are also considered.

The simplifications carried in this section depend on various waveguide parameters and therefore are valid for the quasi-TE mode of a 250 nm-by-550 nm SOI waveguide and similar designs. They also depend on the temperature range achieved with the heaters and the bandwidth of the incoming light, which are considered close to our experimental conditions, namely, temperature excursion lower than 100 K and bandwidth around 10 THz. Nonetheless, our discussion provides a general procedure that is readily applicable to other device designs and operation conditions and allows to identify terms that might need to be incorporated as corrections.

The real electric field of an incoming broadband source can be written as

$$E(t) = \int_{-\infty}^{+\infty} S(\nu) e^{j2\pi\nu t} d\nu \tag{1}$$

where $S(\nu)$ is the Fourier Transform of $E(t)$,

$$S(\nu) = \int_{-\infty}^{+\infty} E(t) e^{-j2\pi\nu t} dt \tag{2}$$

Since $E(t)$ is real, it follows that

$$S(\nu) = S^*(-\nu). \tag{3}$$

Neglecting proportionality constants, the power spectral density can be defined as[6]

$$PSD(\nu) \equiv |S(\nu)|^2. \tag{4}$$

The MZI is composed of input and output couplers with frequency-dependent field coupling coefficients $(t_1(\nu), k_1(\nu))$ and $(t_2(\nu), k_2(\nu))$, respectively, and two arms with attenuation factors $R_1(\nu)$ and $R_2(\nu)$. Thus, after splitting into arms 1 and 2 and recombining at the output waveguide, the electric field that propagated through each arm is

$$E_1(t) = \int_{-\infty}^{+\infty} T_1(\nu) S(\nu) e^{j2\pi\nu t} e^{j\phi_1(\nu)} d\nu \tag{5}$$

$$E_2(t) = \int_{-\infty}^{+\infty} T_2(\nu) S(\nu) e^{j2\pi\nu t} e^{j\phi_2(\nu)} d\nu \tag{6}$$

where $T_1(\nu) = t_1(\nu) t_2(\nu) R_1(\nu)$ and $T_2(\nu) = k_1(\nu) k_2(\nu) R_2(\nu)$ are real transmission factors and $\phi_i(\nu)$ is the real phase accumulated through propagation in arm i, given by

$$\phi_i(\nu) = \beta_i(\nu) L_i = \frac{2\pi\nu}{c} n_{\text{eff,i}}(\nu) L_i \tag{7}$$

where β_i, L_i and $n_{\mathrm{eff,i}}$ are the propagation constant, length and effective index of arm i and c is the speed of light in vacuum. The real nature of the output fields requires that

$$\begin{aligned} &T_i(\nu) = T_i(-\nu) \\ &\phi_i(\nu) = -\phi_i(-\nu) \;\longrightarrow\; n_{\mathrm{eff,i}}(\nu) = n_{\mathrm{eff,i}}(-\nu). \end{aligned} \tag{8}$$

The output power is then obtained time averaging the squared output electric field through many field oscillations,

$$\begin{aligned} I_{\mathrm{out}} &\propto \left\langle E_{out}^2(t) \right\rangle \\ &\propto \left\langle [E_1(t) + E_2(t)]^2 \right\rangle \\ &\propto \left\langle E_1^2(t) \right\rangle + \left\langle E_2^2(t) \right\rangle + 2 \left\langle E_1(t) E_2(t) \right\rangle . \end{aligned} \tag{9}$$

The first two terms in the last row of Supplementary Equation 9 contribute to the mean output power, while the last term contains the interference term of interest. In the following we will focus on manipulating such term, which after some manipulation can be written as

$$I = 2 \left\langle E_1(t) E_2(t) \right\rangle \propto \int_{-\infty}^{+\infty} T(\nu) PSD(\nu) e^{j\Delta\phi(\nu)} d\nu \tag{10}$$

with $T(\nu) = T_1(\nu) T_2(\nu)$ and

$$\Delta\phi = \frac{2\pi\nu}{c} \left[n_{\mathrm{eff},1}(\nu) L_1 - n_{\mathrm{eff},2}(\nu) L_2 \right]. \tag{11}$$

Dropping the proportionality sign and taking I equal to the right hand side (RHS) of Supplementary Equation 10 gives the relation between the oscillatory output power from the MZI – the interferogram – and the PSD in the general case.

The phase difference $\Delta\phi$ is determined by the difference between the optical path ($n_{\mathrm{eff}} L$) of the two arms, as indicated by Supplementary Equation 11. By design the interferometer is balanced ($\Delta\phi = 0$) when no heating is applied, with both arms having the same effective index $n_{\mathrm{eff}}(\nu)$ and arm length L. Typical silicon waveguides present strong dispersion, which are accounted for in a series expansion of the effective index around the central optical frequency ν_0 (193.414 THz in our case) up to third order,

$$n_{\mathrm{eff}}(\nu) = \; n_{\mathrm{eff}}|_{\nu_0} + \partial_\nu n \; \Delta\nu + \frac{1}{2} \partial_{\nu^2} n \; \Delta\nu^2 + \frac{1}{6} \partial_{\nu^3} n \; \Delta\nu^3, \tag{12}$$

using the contracted notation for partial derivatives, $\frac{\partial n_{\mathrm{eff}}}{\partial x} \equiv \partial_x n$ and with $\Delta\nu = \nu - \nu_0$.

In practice, chip-scale variations in the device layer thickness and fabrication imperfections will cause differences between the two arms. These differences, considered small, are introduced as "δ" terms. For instance, the difference in effective index at ν_0 and its first derivative are denoted by $\delta(n_{\mathrm{eff}}|_{\nu_0})$ and $\delta(\partial_\nu n)$ respectively, while the difference introduced in arm length is δL.

When the heaters are actuated, the temperature change ΔT modifies the effective index through the thermo-optic effect and the length of the silicon waveguide arm through thermal expansion. For the Si-FTS, it is important to consider the high order temperature dependence of both effects, as well as the dispersion of the thermo-optic effect. As discussed in the previous section, for temperatures rising up to 100 K above room temperature (i.e. up to ~400 K), it suffices to consider the first and the second order temperature-dependence of the TOC and of the thermal expansion coefficient, respectively. These lead to a second order and third-order temperature dependence of the effective index change Δn_{eff} and

of the arm length change ΔL,

$$\Delta n_{\text{eff}}(\nu,\Delta T) = \left(\partial_T n + \partial_{\nu,T} n\ \Delta\nu + \frac{1}{2}\partial_{\nu^2,T} n\ \Delta\nu^2 + \frac{1}{6}\partial_{\nu^3,T} n\ \Delta\nu^3\right)\Delta T + \tag{13}$$
$$\frac{1}{2}\left(\partial_{T^2} n + \partial_{\nu,T^2} n\ \Delta\nu + \frac{1}{2}\partial_{\nu^2,T^2} n\ \Delta\nu^2 + \frac{1}{6}\partial_{\nu^3,T} n\ \Delta\nu^3\right)\Delta T^2$$

$$\Delta L(\Delta T) = L\left(\ \alpha_1\Delta T +\ \alpha_2\Delta T^2 +\ \alpha_3\Delta T^3\right). \tag{14}$$

Modifications of the effective index due to thermal expansion of the waveguide in its cross-section is negligible compared to the other effects and are not included.

With no loss of generality, we introduce temperature effects in arm 1 while we include the differences of effective index and arm length in arm 2, such that

$$n_{\text{eff},1}(\nu,\Delta T) = n_{\text{eff}}(\nu) + \Delta n_{\text{eff}}(\nu,\Delta T) \qquad n_{\text{eff},2}(\nu) = n_{\text{eff}}(\nu) + \delta n(\nu) \tag{15}$$
$$L_1(\Delta T) = L + \Delta L(\Delta T) \qquad L_2 = L + \delta L \tag{16}$$

with

$$\delta n(\nu) = \ \delta(n_{\text{eff}}|_{\nu_0}) + \delta(\partial_\nu n)\ \Delta\nu + \frac{1}{2}\delta(\partial_{\nu^2} n)\ \Delta\nu^2 + \frac{1}{6}\delta(\partial_{\nu^3} n)\ \Delta\nu^3 \tag{17}$$

The phase difference $\Delta\phi$ can be expressed in an efficient way to sort and evaluate the contribution of the various orders of frequency detuning $\Delta\nu$ and time delay τ introduced by the temperature difference ΔT. Substituting eqs.12-17 into Supplementary Equation 11 and manipulating the resulting expression we write

$$\Delta\phi(\nu,\tau) = \varphi(\nu)\ +\ 2\pi\sum_{i=1}^{5}\frac{\eta_i}{\eta_1^i}\tau^i[\nu_0 +\ \Delta\nu(1+\xi_i)]\left[1 + \left(\frac{\chi_i+\xi_i}{1+\xi_i}\right)\frac{\Delta\nu}{\nu_0} + \left(\frac{\chi_i+\zeta_i}{1+\xi_i}\right)\left(\frac{\Delta\nu}{\nu_0}\right)^2 + \left(\frac{\zeta_i}{1+\xi_i}\right)\left(\frac{\Delta\nu}{\nu_0}\right)^3\right]. \tag{18}$$

The first term, φ, gathers frequency-dependent terms that do not depend on τ. It is given by

$$\varphi(\nu) = -2\pi\ t_0\left[\nu_0\sigma_0 + (\nu_0\sigma_1+\sigma_0)\ \Delta\nu + (\nu_0\sigma_2+\sigma_1)\ \Delta\nu^2 + (\nu_0\sigma_3+\sigma_2)\ \Delta\nu^3 + \sigma_3\ \Delta\nu^4\right] \tag{19}$$

with $t_0 \equiv \frac{L}{c}$ and

$$\sigma_0 = \delta(n_{\text{eff}}|_{\nu_0}) + \frac{\delta L}{L} n_{\text{eff}}|_{\nu_0} \quad \text{and} \quad \sigma_i = \delta(\partial_{\nu^i} n) + \frac{\delta L}{L}\partial_{\nu^i} n \qquad (i=1,2,3) \tag{20}$$

Assuming $\sigma_{-1} = \sigma_4 = 0$, Supplementary Equation 19 can be recast in a compact form as

$$\varphi(\nu) = \sum_{i=0}^{4} t_0(\sigma_{i-1} + \nu_0\sigma_i)\ \Delta\nu^i. \tag{21}$$

With no dependence in τ, φ does not contribute to the kernel of the transformation between time and frequency, on the other hand, it is the only term with contribution from δ-terms.

For now, we focus on the summation containing the *frequency*$\times$*delay* terms of interest. In this form, the summation gathers the remaining terms by increasing order of τ. The terms τ^i are weighted by η_i/η_1^i, and the time delay itself is related to the temperature difference by $\tau = \eta_1\Delta T$. Each η_i gathers

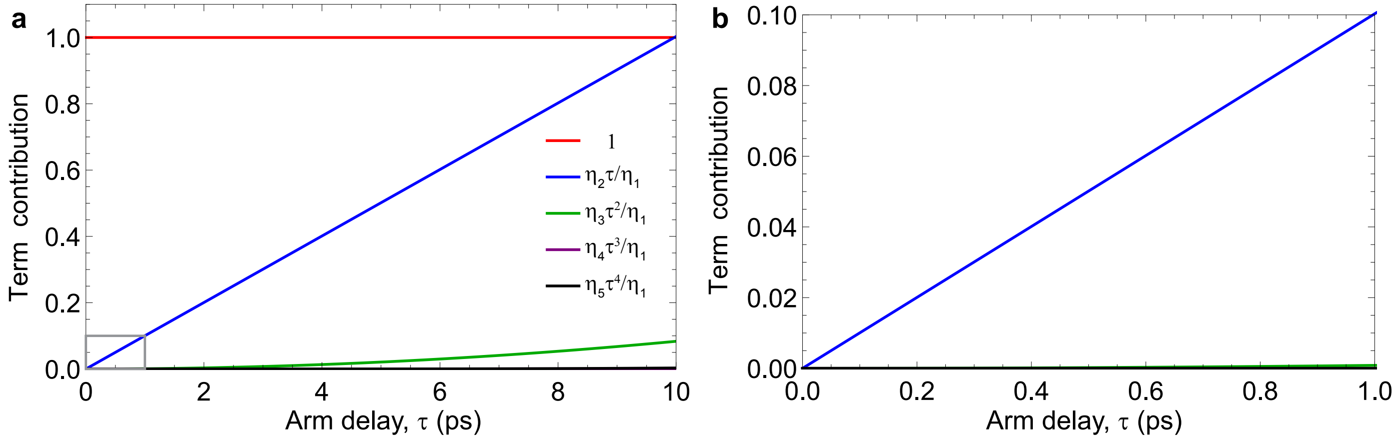

Supplementary Figure 3: Contribution of terms $\eta_i\tau^i/\eta_1^i$ with respect to the first order $\eta_1\tau$ as a function of the time delay. **a.** Range between 0 and 10 ps. **b.** Range between 0 and 1 ps – range of our experiments.

terms in T^{-i}, multiplied by the characteristic time $\tau_0 \equiv \frac{L}{c}$,

$$\begin{aligned}\eta_1 &= t_0(n_{\text{eff}}|_{\nu_0}\ \alpha_1 + \partial_T n),\\ \eta_2 &= t_0(n_{\text{eff}}|_{\nu_0}\ \alpha_2 + \partial_T n\ \alpha_1 + \frac{1}{2}\partial_{T^2} n)\\ \eta_3 &= t_0(n_{\text{eff}}|_{\nu_0}\ \alpha_3 + \partial_T n\ \alpha_2 + \frac{1}{2}\partial_{T^2} n\ \alpha_1)\\ \eta_4 &= t_0(\partial_T n\ \alpha_3 + \frac{1}{2}\partial_{T^2} n\ \alpha_2)\\ \eta_5 &= t_0(\frac{1}{2}\partial_{T^2} n\ \ \alpha_3).\end{aligned} \tag{22}$$

Assuming $\partial_{T^0} n \equiv n_{\text{eff}}|_{\nu_0}$ and $\alpha_0 \equiv 1$, these terms can be written in a general form as

$$\eta_k = \ t_0 \sum_{i=0}^{k} \frac{1}{i!}\partial_{T^i} n\ \alpha_{k-i}. \tag{23}$$

The contribution of high order terms compared to the first order is depicted in Supplementary Figure 3 as a function of the time delay. These plots were obtained using the parameters summarized in Supplementary Table 1. The second order (blue trace) contributes significantly and therefore cannot be neglected even for small delays. The third order term can be neglected for small delays, but it becomes increasingly important for large delays required for high spectral resolution. It reaches 1% of the first order contribution at 3.5 ps, and $\sim$ 10% at 10 ps (Supplementary Figure 3a). Higher order terms are very small and can be neglected even for large delays. In our experiment, we achieve a maximum time delay around 1 ps, in which regime it suffices to keep the first and second order contributions (Supplementary Figure 3b).

Multiplying each order in τ in Supplementary Equation 18 we have terms of increasing order in $\Delta\nu$. The first brackets $[\nu_0 + \ \Delta\nu(1+\xi_i)]$ represent zeroth and first order contributions, while the second brackets assemble higher orders terms. The parameters ξ_i, χ_i and ζ_i are adimensional and represent,

respectively, the first, second and third order dispersion terms in T^{-i}:

$$
\begin{aligned}
\xi_1 &= \frac{t_0\nu_0}{\eta_1}(\partial_\nu n\ \alpha_1 + \partial_{\nu,T} n) &
\chi_1 &= \frac{1}{2}\frac{t_0\nu_0^2}{\eta_1}(\partial_{\nu^2} n\ \alpha_1 + \partial_{\nu^2,T} n) &
\zeta_1 &= \frac{1}{6}\frac{t_0\nu_0^3}{\eta_1}(\partial_{\nu^3} n\ \alpha_1 + \partial_{\nu^3,T} n) \\
\xi_2 &= \frac{t_0\nu_0}{\eta_2}(\partial_\nu n\ \alpha_2 + \partial_{\nu,T} n\ \alpha_1 + \frac{1}{2}\partial_{\nu,T^2} n) &
\chi_2 &= \frac{1}{2}\frac{t_0\nu_0^2}{\eta_2}(\partial_{\nu^2} n\ \alpha_2 + \partial_{\nu^2,T} n\ \alpha_1 + \frac{1}{2}\partial_{\nu^2,T^2} n) &
\zeta_2 &= \frac{1}{6}\frac{t_0\nu_0^3}{\eta_2}(\partial_{\nu^3} n\ \alpha_2 + \partial_{\nu^3,T} n\ \alpha_1 + \frac{1}{2}\partial_{\nu^3,T^2} n) \\
\xi_3 &= \frac{t_0\nu_0}{\eta_3}(\partial_\nu n\ \alpha_3 + \partial_{\nu,T} n\ \alpha_2 + \frac{1}{2}\partial_{\nu,T^2} n\ \alpha_1) &
\chi_3 &= \frac{1}{2}\frac{t_0\nu_0^2}{\eta_3}(\partial_{\nu^2} n\ \alpha_3 + \partial_{\nu^2,T} n\ \alpha_2 + \frac{1}{2}\partial_{\nu^2,T^2} n\ \alpha_1) &
\zeta_3 &= \frac{1}{6}\frac{t_0\nu_0^3}{\eta_3}(\partial_{\nu^3} n\ \alpha_3 + \partial_{\nu^3,T} n\ \alpha_2 + \frac{1}{2}\partial_{\nu^3,T^2} n\ \alpha_1) \\
\xi_4 &= \frac{t_0\nu_0}{\eta_4}(\partial_{\nu,T} n\ \alpha_3 + \frac{1}{2}\partial_{\nu,T^2} n\ \alpha_2) &
\chi_4 &= \frac{1}{2}\frac{t_0\nu_0^2}{\eta_4}(\partial_{\nu^2,T} n\ \alpha_3 + \frac{1}{2}\partial_{\nu^2,T^2} n\ \alpha_2) &
\zeta_4 &= \frac{1}{6}\frac{t_0\nu_0^3}{\eta_4}(\partial_{\nu^3,T} n\ \alpha_3 + \frac{1}{2}\partial_{\nu^3,T^2} n\ \alpha_2) \\
\xi_5 &= \frac{t_0\nu_0}{\eta_5}(\frac{1}{2}\partial_{\nu,T^2} n\ \alpha_3) &
\chi_5 &= \frac{1}{2}\frac{t_0\nu_0^2}{\eta_5}(\frac{1}{2}\partial_{\nu^2,T^2} n\ \alpha_3) &
\zeta_5 &= \frac{1}{6}\frac{t_0\nu_0^3}{\eta_5}(\frac{1}{2}\partial_{\nu^3,T^2} n\ \alpha_3).
\end{aligned}
\tag{24}
$$

They can be presented in a compact form analogous to Supplementary Equation 23 assuming $\partial_{\nu^i,T^0} n \equiv \partial_{\nu^i} n$, $\alpha_0 \equiv 1$ and $\alpha_4 = \alpha_5 \equiv 0$:

$$
\begin{aligned}
\xi_k &= \frac{\nu_0}{\eta_k}\frac{\partial \eta_k}{\partial \nu} = \frac{t_0\nu_0}{\eta_k}\sum_{i=0}^{k}\frac{1}{i!}\partial_{\nu,T^i} n\ \alpha_{k-i} \\
\chi_k &= \frac{\nu_0^2}{\eta_k}\frac{\partial^2 \eta_k}{\partial \nu^2} = \frac{1}{2}\frac{t_0\nu_0^2}{\eta_k}\sum_{i=0}^{k}\frac{1}{i!}\partial_{\nu^2,T^i} n\ \alpha_{k-i} \\
\zeta_k &= \frac{\nu_0^3}{\eta_k}\frac{\partial^3 \eta_k}{\partial \nu^3} = \frac{1}{6}\frac{t_0\nu_0^3}{\eta_k}\sum_{i=0}^{k}\frac{1}{i!}\partial_{\nu^3,T^i} n\ \alpha_{k-i}.
\end{aligned}
\tag{25}
$$

The contribution of the various terms in the second brackets is depicted in Supplementary Figure 4 as a function of the fractional bandwidth $\Delta\nu/\nu_0$. For $\nu_0 = 193.414$ THz, a fractional bandwidth of 0.25, the upper limit of Supplementary Figure 4a, corresponds to a frequency range from 145 THz to 240 THz (wavelength range from 1160 nm to 1930 nm), encompassing the full range of operation of expected Si-FTS designs operating around this ν_0. Even in such large frequency range the contribution of high order terms is overall small, with individual terms reaching a maximum of 5% of the linear term (corresponding to 1). From Supplementary Figure 4a we identify the terms in order $\tau^2\,\Delta\nu^2$ (blue trace), $\tau\,\Delta\nu^3$ (red-dashed trace) and $\tau^3\,\Delta\nu^3$ (green-dashed trace) as those with increasingly significant contribution for large fractional bandwidth, while the other terms are negligible. In our experiments

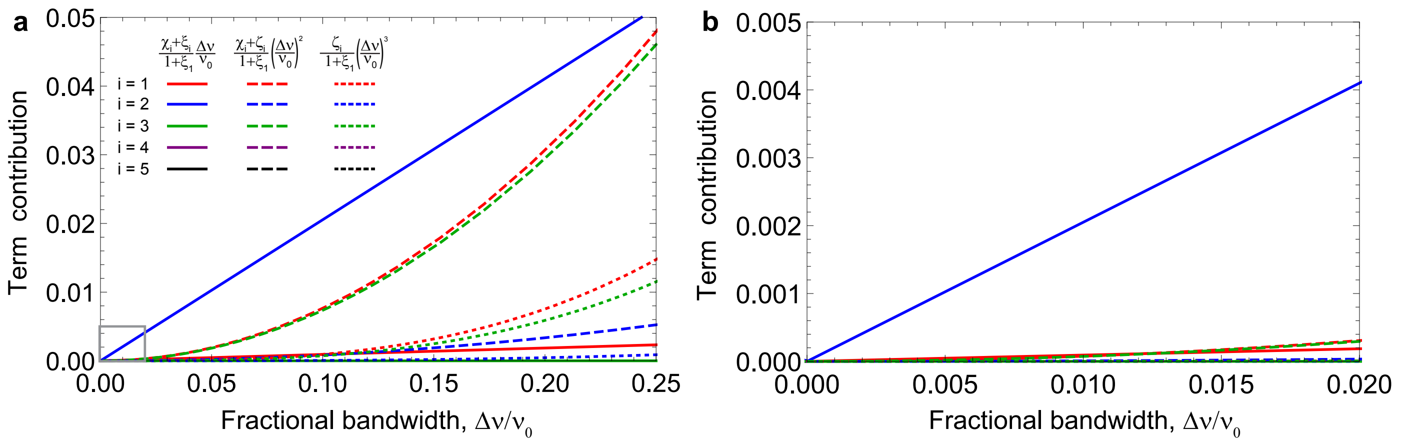

Supplementary Figure 4: Contribution of each term in the second brackets of Supplementary Equation 18 as a function of the fractional bandwidth. a. Range between 0 and 0.25 –large fractional bandwidth. **b.** Range between 0 and 0.02 – range of our experiments.

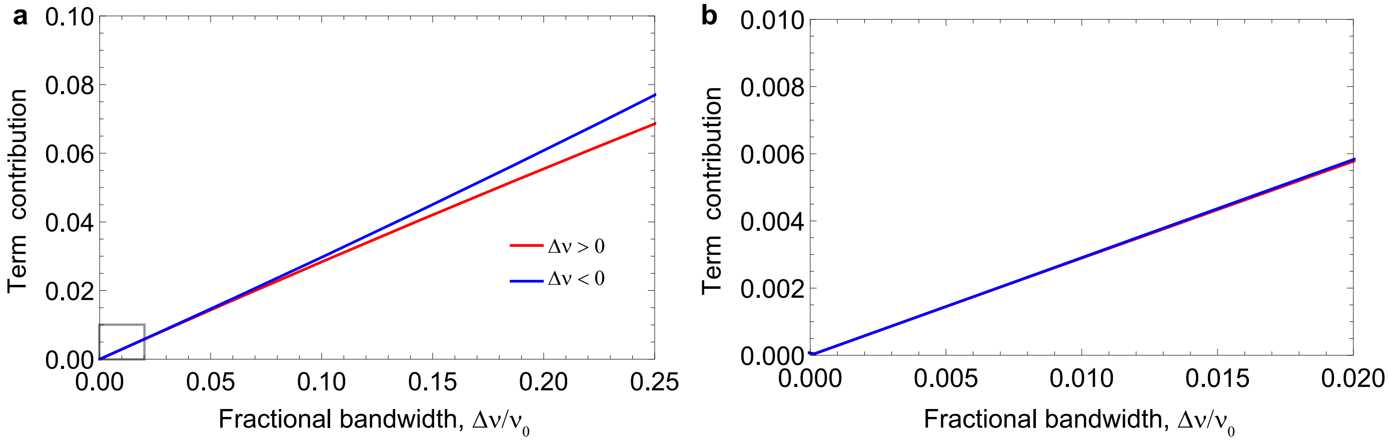

Supplementary Figure 5: Contribution of F_ξ in Supplementary Equation 27 as a function of the fractional bandwidth. a. Range between 0 and 0.25 –large fractional bandwidth. **b.** Range between 0 and 0.02 – range of our experiments.

we used a broadband source with total bandwidth around 7 THz centered at 193.44 THz, with a maximum fractional bandwidth around 0.02. In this case, depicted in Supplementary Figure 4b, all the contributions are smaller than 0.5% and considered negligible.

Based on the these results (Supplementary Figure 3,4), we keep only the first and second order terms in τ and we neglect the high orders in $\Delta\nu$ altogether, leaving only the unity in the second brackets of Supplementary Equation 18.

The phase difference then simplifies to

$$\Delta\phi(\nu,\tau) = \varphi(\nu) + 2\pi\{\tau[\nu_0 + \Delta\nu(1+\xi_1)] + \frac{\eta_2}{\eta_1^2}\tau^2[\nu_0 + \Delta\nu(1+\xi_2)]\}. \tag{26}$$

Defining $\gamma_2 \equiv \eta_2/\eta_1^2$ and rearranging the expression,

$$\Delta\phi(\nu,\tau) = \varphi(\nu) + 2\pi\left\{\left[\tau + \gamma_2\tau^2(1+F_\xi)\right]\left[\nu_0 + \Delta\nu(1+\xi_1)\right]\right\}, \tag{27}$$

with

$$F_\xi = \frac{(\xi_2 - \xi_1)\frac{\Delta\nu}{\nu_0}}{1 + \xi_1\frac{\Delta\nu}{\nu_0}}. \tag{28}$$

Once again we have an adimensional parameter F_ξ to be compared to 1 as a function of the fractional bandwidth (Supplementary Figure 5). F_ξ is slightly different depending on the sign of $\Delta\nu$ as indicated in Supplementary Figure 5a and reaches a maximum around 8% at $\frac{\Delta\nu}{\nu_0} = 0.25$, at which point corrections related to high order terms in $\tau\ \Delta\nu$ might also be considered as previously discussed. Close to our experimental conditions, however, the term is smaller than 1% (Supplementary Figure 5b) and can be neglected.

Finally, the phase difference assumes the simple form

$$\Delta\phi(\nu,\tau) = \varphi(\nu) + 2\pi u\mathcal{T}, \tag{29}$$

with

$$\begin{aligned} \mathcal{T} &\equiv \tau + \gamma_2\tau^2 \\ u &\equiv \nu_0 + \Delta\nu(1+\xi_1) \end{aligned} \tag{30}$$

as modified time delay and optical frequency. $\mathcal{T}$ linearizes the delay metrics, while u stretches the original frequency ν around ν_0 by a factor $(1+\xi_1)$.

Substituting eqs.29,30 in Supplementary Equation 10 and performing the change of variables from ν to u in the integral,

$$\begin{aligned} I(\mathcal{T}) &= \frac{1}{1+\xi_1}\int_{-\infty}^{+\infty} T(u)PSD(u)e^{j\varphi(u)}e^{j2\pi u\mathcal{T}}du \\ &= \frac{1}{1+\xi_1}\mathcal{F}\left[T(u)PSD(u)e^{j\varphi(u)}\right] \end{aligned} \tag{31}$$

where $\mathcal{F}\left[\ \right]$ denotes the Fourier Transform. Neglecting the constant term $(1+\xi_1)^{-1}$ multiplying the FT, the PSD is then retrieved from the absolute value of the inverse-FT of the interferogram, normalized by the MZI transfer function $T(u)$,

$$PSD(u) = \frac{\left|\mathcal{F}^{-1}\left[I(\mathcal{T})\right]\right|}{T(u)}. \tag{32}$$

Finally, the frequency axis must be transformed back to the original optical frequency ν,

$$PSD(u) \xrightarrow{\nu=\frac{u-\nu_0}{1+\xi_1}+\nu_0} PSD(\nu). \tag{33}$$

Supplementary References

[1] Frey, B. J., Leviton, D. B. & Madison, T. J. Temperature dependent refractive index of silicon and germanium. In *Proc. SPIE, Optomechanical Technologies for Astronomy*, vol. 6273 II, 62732J (2006).

[2] Leviton, D. B. & Frey, B. J. Temperature-dependent absolute refractive index measurements of synthetic fused silica. In *Proc. SPIE, Optomechanical Technologies for Astronomy*, vol. 6273 II, 62732K (2006).

[3] Della Corte, F. G., Esposito Montefusco, M., Moretti, L., Rendina, I. & Cocorullo, G. Temperature dependence analysis of the thermo-optic effect in silicon by single and double oscillator models. *Journal of Applied Physics* **88**, 7115–7119 (2000).

[4] Li, H. H. Refractive index of silicon and germanium and its wavelength and temperature derivatives. *Journal of Physical and Chemical Reference Data* **9**, 561–658 (1980).

[5] Okada, Y. & Tokumaru, Y. Precise determination of lattice parameter and thermal expansion coefficient of silicon between 300 and 1500 K. *Journal of Applied Physics* **56**, 314–320 (1984).

[6] Yariv, A. *Optical electronics* (Rinehart & Winston, New York, 1985), 3 edn.
